# Events in context—The HED framework for the study of brain, experience and behavior

**DOI:** 10.3389/fninf.2024.1292667

**Published:** 2024-05-23

**Authors:** Scott Makeig, Kay Robbins

**Affiliations:** ^1^Swartz Center for Computational Neuroscience, Institute for Neural Computation, University of California San Diego, La Jolla, CA, United States; ^2^Department of Computer Science, University of Texas San Antonio, San Antonio, TX, United States

**Keywords:** HED, Hierarchical Event Descriptors, event, context, neuroimaging, BIDS, data standards, analysis-ready data

## Abstract

The brain is a complex dynamic system whose current state is inextricably coupled to awareness of past, current, and anticipated future threats and opportunities that continually affect awareness and behavioral goals and decisions. Brain activity is driven on multiple time scales by an ever-evolving flow of sensory, proprioceptive, and idiothetic experience. Neuroimaging experiments seek to isolate and focus on some aspect of these complex dynamics to better understand how human experience, cognition, behavior, and health are supported by brain activity. Here we consider an event-related data modeling approach that seeks to parse experience and behavior into a set of time-delimited events. We distinguish between *event processes* themselves, that unfold through time, and *event markers* that record the experiment timeline latencies of event onset, offset, and any other event phase transitions. Precise descriptions of experiment events (sensory, motor, or other) allow participant experience and behavior to be interpreted in the context either of the event itself or of all or any experiment events. We discuss how events in neuroimaging experiments have been, are currently, and should best be identified and represented with emphasis on the importance of modeling both events and event context for meaningful interpretation of relationships between brain dynamics, experience, and behavior. We show how text annotation of time series neuroimaging data using the system of Hierarchical Event Descriptors (HED; https://www.hedtags.org) can more adequately model the roles of both events and their ever-evolving context than current data annotation practice and can thereby facilitate data analysis, meta-analysis, and mega-analysis. Finally, we discuss ways in which the HED system must continue to expand to serve the evolving needs of neuroimaging research.

## 1 Introduction

The human brain has evolved to continuously optimize the results of its active cognition, including the behavior it controls, by taking into account anticipated and perceived challenges and opportunities. To optimize our behavior (and attentional focus), the brain must hold, update, and respond effectively to ever-changing expectations. These are shaped by relationships of our recent/ongoing experience (our active present) to our longer-term aims and needs, and by our co-evolving awareness of their active constraints and prerequisites. Because our lived environment is so complex—including, importantly, the variable predictability of the behavior of other agents—our behavioral decision-making must account for our awareness of ever-evolving experienced (past) and anticipated (future) contingencies on multiple space and time scales, from fractions of a second to years (or even millennia in the case of cultural traditions and limitations). This complexity of human experience and action within our dynamic human life context makes the study of human brain dynamics supporting experience and behavior most challenging.

At the same time, recording concurrent brain and behavioral data is ever more feasible in static as well as concurrent brain and body imaging paradigms that involve motivated actions performed in 3D environments. This paper proposes a conceptual framework for describing and recording events and their contexts in a way that can capture this complexity. We show how the emerging system of Hierarchical Event Descriptors (HED; https://www.hedtags.org) and the tool infrastructure built on this framework can be used to construct concrete, analysis-ready, human- and machine-actionable descriptions of events occurring during acquisition of time-series neuroimaging data of any modality (EEG/MEG, fMRI, etc.) to support search, summary, and analysis of the recorded brain activity, within or across studies.

### 1.1 Articulating our experience

How we remember, recall, and describe our experience tends to be structured as a series of temporally separate, contiguous, or overlapping events unfolding on different time scales. Our memory for and communications about our unfolding experience highlights the role of experiential boundaries (Shin and DuBrow, 2021). For example, the question, “What did you do on your recent business trip?,” posed to a colleague, might prompt a response such as: “I drove to the airport at [time] to catch the plane to [place], checked into [hotel], walked to [restaurant] to eat dinner, went to bed [early], slept [well], ate [a full breakfast] to prepare myself to [work activity] ….” Here the responder recounts their (recalled or recorded) “trip event” as a series of shorter duration (though not necessarily temporally distinct or contiguous) experienced and/or performed *event processes*.

The same responder might also recall each recounted trip event and its defining *event phases* (including its temporal *event boundaries*) as a sequence or more temporally general collection of shorter duration events (*event processes*) and respective boundary phases, each possibly defined by some aspects of their experience. For example, the responder might have described their travel experience in terms of experienced feeling changes: “I left home feeling [slightly anxious], and didn't begin to feel [confident] about [doing what I needed to do] until [the morning] when I felt [relieved] to realize that ….” Event-wise annotation of an emotional timeline for the same trip might thus require a different set, stream, or tier of event period descriptions and contrastive event boundaries. A still more complete annotation of the trip might comprise further event streams or tiers, for example phenomena noted *post hoc* in recorded psychophysiological data streams (changes in respiration, sweating, heart rhythm variability, etc.).

Thus, answering the simple question, “What happened?,” during a neuroimaging recording in a way capable of supporting planned and/or possible further analyses of the combined data record requires a flexible, robust, and extensible annotation system.

### 1.2 Event processes and phases

While we would likely agree that time passes as a continuous flow, if we are asked to attempt to fully experience this continuity we may likely focus on attending to some contiguous series of event process phase boundaries (breath inhalations and exhalations, circular sweeps of a clock hand, etc.), and tend to group these into a sequence of events (breath cycles, hours), similar to how we regularly perceive a train of identical isochronous clicks as a sequence of duple *tick-tock* events, each lasting two ticks (Nozaradan et al., 2011).

The tendency to separate continuous experience into discrete events is studied in psychology as event segmentation (Zacks et al., 2007). Sasmita and Swallow (2023) have reported the large degree of agreement on event segmentation boundaries across groups, supporting the use of event and event phase segmentation to map recordings of continuous experience into event processes for analysis. Evidence has also been presented showing that the parsing of ongoing activity into events with discrete boundaries is involved in the updating of working memory and of learning (Kurby and Zacks, 2008).

Thus, we typically recall and recount our experience in terms of *event phase transitions* between successive periods of coherent experience or action. Here we refer to experienced events as *event processes* to emphasize their inherent temporal extent. For example, we may recount our experience in viewing a long movie as a single experienced (movie watching) event or *event process* but may then recall our experience in more detail by referring to disjunctive (and thereby memorable) moments within it. These points of *event phase transition* include its own temporal boundaries (*onset* and *offset*) as well as (*inset*) moments within it that either bound successive, briefer periods of more coherent experience (for example: individual story scenes or camera shots), or else mark critical points within these (for example, sensory and/or dramatic climaxes within a scene or shot). Other events or event processes experienced during movie viewing may not coincide with these cinematic (sub)events—for example, feeling hungry or thirsty during some portion of the movie watching experience or walking out to the lobby to buy snacks.

During experiments recording brain (and/or behavioral) time series data to study human cognition, questions of key interest typically concern how brain activity and/or behavior represent our anticipation of or response to experienced events—presented sensory stimuli, required actions, and/or task-related decisions. Event-related analysis of brain time series data requires that these perceptual, behavioral, and cognitive (task-condition related) events (event processes) within the experience of experiment participants be recorded and described in a form suitable for subsequent analysis.

### 1.3 Historical treatment of events

The first experiments to systematically study human behavior and cognition as dynamic processes, those of Donders in the early 1860s, measured upper limits to the speed of manual responding to suddenly presented visual stimuli or of verbally responding to brief presented sounds (described in Goldstein, 2020). To make accurate measurements, these experiments used image presentations with sudden (tachistoscopic) onsets (Volkmann, 1859; Benschop and Draaisma, 2000). Manual response delay distributions (a.k.a. reaction times) produced under different experiment conditions became the primary measure used in the field of mental chronometry, later broadened to experimental psychology and then to cognitive science, a forerunner and behavioral partner to cognitive neuroscience (Jensen et al., 2006).

Unfortunately, in this process the behavioral event of pressing a finger button in response to some task demand came to be treated as, in effect, an “event without duration” defined only by a single event phase transition—switch closure latency with respect to the onset of the preceding imperative stimulus. This conception ignores the complex and temporally extended brain and motor (i.e., psychophysiological) processes involved in the act. Nonetheless, this practice began the pattern of recording, in neuroimaging experiments, only the times of onset for a small set of task-relevant event types (experienced or produced) identified only by numeric codes (as in, “Event type: 213”). This practice rests on an incomplete conceptual framework for describing events that, while forced by the limitations of early recording technology—still dominates time series neuroimaging research. Experiment control applications used in EEG, MEG, fMRI and other time series neuroimaging today often only record these two pieces of information for each event identified in the data record (a non-standard event type code, and an onset latency). Further, standards still in place for data storage and sharing continue to expect non-standard integer event type codes that are often poorly or not at all documented in archived or publicly shared data.

### 1.4 Observing natural cognition

Advancing brain activity imaging technology is now capable of capturing brain activity in ever more detail during a widening range of human behavior and experience. At the same time, macro- and meso-scopic patterns of electromagnetic brain activity accessible through neuroimaging are increasingly seen to modulate effects of concurrent brain activity at smaller spatial and temporal scales, and thereby to play important functional roles in brain dynamics (Pinotsis et al., 2023). Because of the fine time resolution of brain electromagnetic signals (sufficient to record dynamics supporting individual thoughts and actions), EEG and MEG research long focused primarily on statistical perturbations in brain (or scalp) signals following and/or preceding sensory and other events experienced by participants in neuroimaging experiments.

The last 15 years have seen increasing interest in capturing brain dynamics supporting many forms of more natural cognition (Gramann et al., 2014). Research paradigms here involve active perception of complex naturalistic stimuli unfolding through time (speech, music, movies, and animations) and may incorporate measures of visual inspection. They may measure participant movements in unconstrained 3D environments, and/or record interactions between multiple participants. An increasing number of such studies record eye and body movements as well as EEG (and/or fNIRS) data from participants performing more complex task-involved motor actions, or during natural social interactions, either within or outside the laboratory. A broad experiment framework and term for this—mobile brain/body imaging (MoBI)—was first proposed by one of us 15 years ago (Makeig, 2009; Makeig et al., 2009). Virtual- and augmented-reality experiment designs, movie- or annotation-viewing and videogame playing experiments also provide new challenges that will require continued development of event annotation methods as well as data recording (Kothe et al., 2012, 2024) and analysis approaches (Logie and Donaldson, 2021).

### 1.5 Events in fMRI recordings

In recent years, event-related analyses of fMRI and related metabolic imaging data have been less dominant than in EEG and MEG neuroimaging research, as the ~10x or more slower time course and lower sampling rate of BOLD and other MR-related metabolic measures make brain dynamics associated with individual experiment events in metabolic imaging studies more difficult to study. This has contributed to the widespread adoption of the resting state task paradigm in fMRI studies. Nonetheless, slower changing BOLD and/or other metabolic signals recorded by functional imaging methods must also integrate slower changes in brain/body activity linked to participant experience of event processes occurring on faster time scales, before and during recording as well as any events participants may anticipate occurring following the recording period. Now, techniques such as phase-encoded fMRI (Engel, 2012) allow sub-second resolution of certain types of ongoing processes, making the proper documentation of events on all time scales even more important for fMRI studies. As well, participant characteristics can be influential in determining brain state and dynamics and can be modeled as event processes whose time evolution is constant with respect to the recording and is thereby handled using the same computational framework as event processes within the experience of the participant.

### 1.6 Event-related data analysis

Berger's first EEG reports (from 1926) noted changes in the scalp-recorded signals during different states including sleep, eyes open vs. closed, and arithmetic task performance—as no doubt recorded by hand (Ince et al., 2020). The strong focus on reaction-time studies in experimental psychology provided the ground for the ready adoption of the event-related data analysis approach in psychology laboratory EEG (and later, other modality) studies. The first computer system for measuring EEG responses to (repeated) presentations of (identical) sensory stimuli, the *Computer of Average Transients (CAT)* of Clynes (1962), computed (by analog means) average EEG responses time locked to onsets of some set of stimulus presentation events (based on the assumption that they should produce equivalent effects on brain activity). In early computerized systems built to control and record EEG experiment sessions, event types were signaled by synchronous TTL “trigger” pulses sent out on some subset of lines in the parallel port bus. Originally, these literally triggered analog signal generation devices to present auditory or visual stimuli, though the analog terminology remains in use in many laboratories today.

The resulting event-related EEG time series averages were originally termed trial-averaged *Evoked Potentials (EPs)* (Dustman and Beck, 1963). Soon, however, it was pointed out that omissions of anticipated stimuli also produced mean event-related potential fluctuations, so the more inclusive term *Event-Related Potentials (ERPs)* (Treisman, 1960) came to dominate. ERPs dominated psychophysiological studies for the next half century, serving as the first functional brain imaging modality (though imaging was generally confined to plotting the computed response time course for single scalp channels). In recent decades, event-related EEG (and corresponding magnetoencephalographic, MEG) analysis methods have been extended to include the time/frequency domain (Makeig, 1993; Makeig et al., 2002), inverse cortical source imaging (Van Veen and Buckley, 1988; Makeig et al., 1995), and cross-frequency and cortical network (Delorme et al., 2011; Martínez-Cancino et al., 2019) analysis. These approaches can extract more information about interrelationships between experienced events and cortical brain dynamics than is available in scalp-channel ERPs alone. Similarly, current fMRI research has progressed beyond simple (task block minus rest block) BOLD signal measures to studies of coherence in (mostly) spontaneous BOLD signal patterns using network models (Wang et al., 2021).

It seems high time that descriptions of participant experience during neuroimaging experiments should also progress beyond the simple (event type and onset latency) framework now in near universal use in the field, in part continuing approaches adopted by the earliest computerized data recording systems. In constructing the current (3rd) generation of the HED event description system described in this overview article, we have attempted to provide a more complete framework in which each recorded experiment event should be associated with an explicit event period demarcated by two event phase boundaries—its *onset* and *offset* moments—as well as any other intermediate *(inset)* phase transition moments of current or possible future analysis interest. To retain compatibility with current-practice experiment records, HED tools for event search, summary and analysis also accept this information in the form of event onset latencies and durations, internally building and then operating on a table of event processes and associated event phase markers.

### 1.7 Event context

A primary interest in event-related neuroimaging studies is their disclosure of differences in brain processing before, during, and following events depending on their perceived cognitive significance, as represented by response differences to different classes of experiment stimuli under different task instructions or sensory or task load conditions. Analysis methods that simply produce mean response statistics treat variability within the collection of averaged trials essentially as noise, ignoring the effects of variability in expectation and priming produced by immediately preceding events—for example, the number of statistically rare target type stimuli that immediately precede presentation of another target stimulus in an oddball task sequence (Squires et al., 1977). Event-related response variations appear in response to differences in the *event context* in which a given event unfolds. An efficient system for analysis of event-related brain and/or behavioral data should therefore record, and make readily available for analysis, the concurrent and precedent event induced context in which each recorded event is experienced by the participant(s)—a goal of the current HED system.

Event annotations should also be human- as well as machine-readable. To enable application of efficient analysis pipelines to larger collections of well-annotated *analysis-ready* data, its accompanying metadata including event annotations must use standardized vocabulary and syntax, making the recorded events *and their respective experiential contexts* machine-actionable. Event records should be sufficiently complete to avoid the need for custom data handling and laborious “event detail sleuthing.”

In any system, therefore, event-handling requires a software tool infrastructure that must have (at least) the following capabilities:

1. Ability to identify and locate *experiment events* whose natures satisfy complex search criteria (expressed in a dataset independent manner using standardized vocabulary and syntax).
2. Ability to derive and identify *event contexts* from events concurrent to each event process and event phase marker.
3. Ability to inform the analysis with task-related or other relationships between current and *preceding* or *succeeding events*.

In the following sections, we consider the nature of events in neuroimaging data and the value of studying event-related brain dynamics within their individual experiential context. We then show how the HED structure, vocabulary, and software tool infrastructure can be used to study relationships between human behavior, experience, and brain dynamics in both classic psychophysiological experiment paradigms and in contemporary paradigms involving continuous flows of perception, action, and cognition. We begin by defining the meaning of the term *event* in the HED framework.

## 2 Experiment events

We here define an experiment event simply as “something happening” (i.e., some process unfolding through time), whose phases and details are experienced as belonging to a single process that occurs (i.e., that happens, takes place, is perceived, or is performed, etc.) during some recorded time period during the experiment. This definition covers a variety of alternative definitions in the fields of philosophy, probability, physics, and computer science. Here an event (or event process) refers to an identifiable, temporally-demarcated experience or action of a participant during a neuroimaging experiment—or else, any time-limited feature noted in the experiment data record itself (whether identified during or after recording).

Our definition implies these characteristics of experiment events:

1. An *event* (or *event process*) is an identifiable process that unfolds through some period of time on the (single) data recording timeline.
2. The nature and attributes of an event process are identifiable and describable.
3. An event process has distinct and known times of *onset* and *offset* on the experiment timeline. These are termed *event phase markers* (or less formally, *event markers*) of the event process.
4. Event markers of an event process may also include intermediate (*inset*) markers of moments of event process phase transition (from strengthening to weakening stimulation, from ballistic to guided movement, from one camera view to another, etc.).
5. Events identified in neuroimaging experiments may be temporally isolated, overlapping, or concurrent, and may occur within the time span of other events. Events may be organized or described as belonging to one or more *event tiers, streams*, or *collections* having roles or properties in common.

Importantly, we distinguish the *event process* itself from its *event phase markers*. This conceptual distinction is the foundation of the HED event annotation system, while in current practice the two are not clearly distinguished. For example, BIDS files recording events occurring during a recording (with filenames ending in *_events.tsv*) typically record an *onset* event marker latency and event type code in each file table entry (row). BIDS also requires a *duration* to be associated with each event marker. However, in practice, event markers often represent entire trials, and the *duration* column is unused or what the duration pertains to (usually not the trial) is undocumented. As mentioned above, current HED tools create a more complete representation of event processes internally to support effective event search, summary, and event-related data analysis.

### 2.1 Event context

The current Google online large language model (LLM) search tool application suggests the meaning of “event context” in the field of philosophy to be, “the sum of all events located within its time location, including the event itself.” More generally, event context may include the “setting” of an event, as described using terms that allow it to be recognized and assessed, and that may extend beyond the temporal boundaries of the event itself. As noted earlier, both brain dynamics and behavior associated with an event may be influenced by its *context* including any or all preceding, accompanying, and/or anticipated events. Thus, it is important that the *event context* in which each experiment event occurs be readily recoverable from the event record.

At least five types of event context are relevant for neuroimaging analysis: recording context, priming and preceding event context, ongoing event context, and imperative event context. All of these can affect imaging measures—and thus merit clear annotation in the experiment record.

#### 2.1.1 Recording context

Many facts characterizing the neuroimaging recording, considered as itself an encompassing experiment event, may affect the neuroimaging data. These include facts concerning the physical environment (time of day, temperature, background noise, and luminance levels, …), the setting (indoors/outdoors, environment stress level). Importantly, they include relevant parameters of the data acquisition process itself (sampling rate, locations of sensors). Both traits and states of experiment participant(s) themselves may also strongly influence performance and brain dynamics, as can relationships *between* participants in multi-participant experiments. Further information, such as the pose of the participant(s) and whether movement was constrained, may also prove relevant.

#### 2.1.2 Priming event context

Priming event context refers to participant experience preceding data collection that may prime the cognitive state or attitude of participants, for example experiments that stage pre-experiment scenarios to affect a participant's mental state (Raz et al., 2002; Henson, 2003). The priming event context thus includes any initial setup, consent, and training processes that a participant undergoes before data collection begins.

#### 2.1.3 Preceding event context

Context produced by events preceding a given experiment event may affect cognitive and behavioral appraisal of and responses to succeeding events, whether the task framework references them. A well-known example is the gambler's fallacy (Tversky and Kahneman, 1974) under which the gambler believes that earlier events affect subsequent events, even when the events are by known design independent.

#### 2.1.4 Ongoing event context

Every current event process contributes to the ongoing event context at each point of the experimental timeline, for example if a still or moving image is being presented when a participant presses a button.

#### 2.1.5 Imperative event context

Imperative event context refers to events whose occurrence affects subsequent events (e.g., task-related participant actions) or their anticipation. Task action imperatives motivating participant actions provide a framework for interpreting participant actions in terms of task goals and constraints.

## 3 The HED approach

The need for systematically recording answers to, “*What happened (exactly)?*,” for each neuroimaging experiment was recognized (presciently) nearly 15 years ago by Nima Bigdely-Shamlo, then a graduate student in the first author's laboratory at UCSD, who then demonstrated a first version of a system of *Hierarchical Event Descriptors (HED)* for describing events using a common vocabulary and syntax (Bigdely-Shamlo et al., 2013). Initial design goals were to make HED annotations both human and machine readable and to avoid as much as possible the user-facing complexities of formal ontologies and databases.

Since its initial inception, HED (now in its third generation) has undergone major conceptual and technical development and has evolved an extensive ecosystem of tools and vocabularies (Robbins et al., 2022) designed to describe the range of lived experience during neuroimaging experiments outlined in Section 2. Interestingly, HED remains the only system developed to describe in detail, using a well-defined but easily extended vocabulary and syntax, “*What happened*?” during a neuroimaging experiment. HED thus became (in 2019) the first and still only event description system accepted by the Brain Imaging Data Structure (BIDS) specifications for formatting stored and shared neuroimaging data (Gorgolewski et al., 2016; https://bids.neuroimaging.io), and is now (2024) under consideration for acceptance as an international standard by the International Neuroinformatics Coordinating Facility (INCF, https://www.incf.org).

During the past 5 years, the HED infrastructure and supporting tools have improved significantly. HEDs mission has been expanded from providing basic vocabularies for annotating events to being a framework for mapping contextual and other information onto an experimental timeline to enable and facilitate event-related search and analysis. HED has a formal specification document in which assertions about the behavior of HED are linked to the error codes to be raised by tools, should those assertions fail. Each error code is associated with a set of unit test cases expressed in JSON that tools such as HED validators should automatically run as part of their automated GitHub workflows.

### 3.1 The HED ecosystem

**Figure 1:**
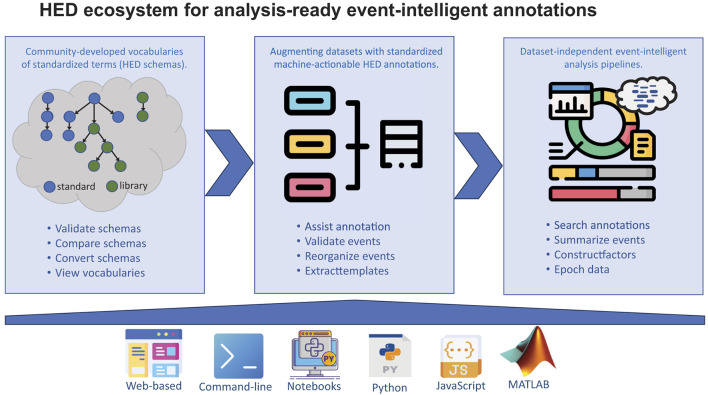
An overview of the HED ecosystem and tools. **Top row:** Left panel: Processes/tools supporting development of standardized HED schemas, which are hierarchically structured vocabularies of terms referred to as *HED tags*. The HED ecosystem has support tools for validating, comparing, converting, and viewing vocabularies to assist communities in developing their own vocabularies. Middle panel: Processes/tools for adding, vetting, and organizing HED event annotations to make data analysis-ready. Right panel: Processes/tools supporting use of HED event annotations in data analysis including sophisticated event-level searching, summarization, and computing factor vectors based on HED tags. **Bottom row:** Tool platforms that support the use of HED. See Supplementary material for examples and Supplementary Table 1 for primary links to resources (icons from https://flaticon.com and https://mathworks.com).

Effective event annotation and handling requires both standardization of annotation vocabulary and syntax, as well as good tools to validate, explore, summarize, and apply event records toward research purposes. The HED tool ecosystem, as illustrated in Figure 1, consists of extensible vocabularies and tools for creation, integration, and use of annotations to support analysis of imaging and behavioral data.

#### 3.1.1 HED schemas

HED schemas are community-developed, heterarchically organized vocabularies (i.e., collections of HED tag term hierarchies, as depicted schematically in the left panel of the top row of Figure 1). Terms that appear deeper in a HED tag hierarchy must be more specialized versions of their parent terms (i.e., each child node term must have an *is-a* relationship to its parent node term). This organization supports search abstraction, essential for effective within- and cross-study event-related data search and analysis. For example, searches for event HED-annotations containing the term *2D-shape* may return event annotations ending either with *2D-shape or* with any of its schema-defined child terms (e.g., *Arrow, Cross, Circle, Triangle*, etc.)—or with any other relevant child term introduced into an event annotation while not currently in a HED schema (here, for example, *2D-shape/Star, 2D-shape/Pentagon*, etc.). The individual tags in a HED schema are uniquely identified, and a goal of the heterarchical organization is that the terms in each tree are disjoint (orthogonal) from those in other trees. See Supplementary Table 2 for examples of simple HED tag searches, and Supplementary Figure 1 for examples of design matrix extraction from HED annotations.

The HED Standard schema (current version searchable at https://www.hedtags.org/display_hed.html) includes several useful tags for identifying and standardizing annotation of environment and other metadata. References to terms in standard ontologies and atlases can be included using tags from the *Metadata* tag subtree to specify source names and unique IDs for the corresponding terms. The HED *Description* tag allows annotators to include additional plain-text information in any annotation. HED tools can either filter out or, if desired, reference this information during event search.

HED has rules for grouping tags using parentheses for expressing associations and complex relationships. The tag string (*Red, Triangle*) refers to a red triangle. Sentence structure is generally expressed as *(subject, (predicate, direct object))* so a participant moving their left hand is usually encoded as (*Experiment-participant*, (*Move*, (*Left, Hand*))).

An important feature of HED is its ready extensibility. When annotating events in a dataset, users may further extend most branches of a tag hierarchy beyond their schema-defined depth, thereby accommodating more specific description while not interfering with more general searches. For example, consider an experiment where the subject is asked to distinguish between apples and oranges in presented images. Subsequent analyses of this dataset might want to find presentations of images of any fruit, within or across available datasets. The HED Standard schema currently represents food and drink items only as *Item/Object/Ingestible-object*. Nonetheless, in their event annotations users might label an *Object* in a presented image as an *Ingestible-object/Apple* or *Ingestible-object/Fruit/Apple*. They may also use HED *Label* syntax for the same purpose (see the HED tutorials, accessible online through https://www.hedtags.org). These approaches allow users to specify (one-off) event details using terms not yet formally incorporated into a validated HED schema.

The process to formally add new (individual) tags to the standard schema requires discussion and, ultimately, voting by the HED Working Group, as strict version control for HED schemas is required to maintain the function of HED tools. Accumulating extension use cases may also incite the development of a HED library schema defining a more complete set of terms necessary to describe experiment events in a specialized research field or subfield (language, music, movement, etc.).

#### 3.1.2 HED libraries

An important HED system feature is its support for formally introducing (sub)field-specific sets of new HED terms in the form of a recognized *HED library schema*. Currently, the *HED_SCORE* library schema (Pal Attia et al., 2023) adds to HED a version of the SCORE vocabulary accepted internationally for annotating clinical EEG recordings by clinical neurophysiologists (Beniczky et al., 2013, 2017). Similarly, a soon to be introduced LANG schema will specify vocabulary needed to sufficiently describe events in experiments on language processing (e.g., specialized terms such as participle, phoneme, etc.). Library schema term specifications include both their plain-text definitions and an indication of where HED event search and summarization tools during operation should attach them to the HED Standard schema (as illustrated in Figure 1 left panel).

#### 3.1.3 HED and ontologies

From its inception, HED development has attempted to evade the complexities associated with using formally enumerated terms organized into formal ontologies for practical annotation. However, there is a direct mapping of HED into a formal ontology using subclass and dis-jointness relationships to enforce the HED vocabulary requirements. A draft ontology and support tools for mapping and workflow implementation are under development. A goal is to be able to link HED annotations with existing ontologies and provide more detailed provenance while preserving HED's role as a user-facing technology.

#### 3.1.4 HED annotations for event files

A standard way to create HED annotations for a BIDS dataset is to use online HED tools to build a template JSON sidecar file listing the unique event-type codes in the dataset event files (names ending in _*events.tsv*). Users fill in event code descriptions and tags, resulting in an annotated dataset. Supplementary Figure 2 illustrates how a JSON event description file template can be extracted using the HED online tools. HED tools include a GUI-based tool, *CTAGGER*, to assist users selecting appropriate HED terms during event annotation. HED python tools also provide numerous facilities through the HED remodeling tool interface to reorganize or modify the columns and or rows of BIDS or other tabular event listing files to enable better reporting and annotation, as illustrated in Supplementary Figure 3. Supplementary Figure 4 gives an overview of the process of using HED tools in performing event-related analysis of BIDS-formatted datasets.

#### 3.1.5 Analyzing HED-tagged data

When invoked by a user, HED event search and summary tools assemble complete, context-enriched annotations for each recorded dataset event, then search the complete event annotations whose events satisfy a wide range of possible search criteria—criteria that may include any category or categories of event context (see Supplementary Table 2 for examples of HED searches.). HED-based event searches and summaries can be applied across one or more HED-annotated datasets. Event summary and visualization tools are available in the OpenNeuro archive (Gorgolewski et al., 2017; https://openneuro.org) via its electrophysiological data portal NEMAR (Delorme et al., 2022; https://www.nemar.org). These include a tool returning a “word cloud” image featuring most often used terms in the input dataset(s) event HED strings. Factor vectors and experiment design matrices can be automatically extracted from well HED-annotated event files, and software such as EEGLAB (Delorme et al., 2011) can use these factors to identify data epochs of interest for analysis. EEGNET (Rogers et al., 2023; https://eegnet.org), a Canadian EEG data portal, supports interactive HED annotation of artifacts and other features in its archived EEG recordings (Supplementary Figure 1 illustrates different methods of design matrix extraction using HED).

### 3.2 HED event context mechanisms

**Figure 2:**
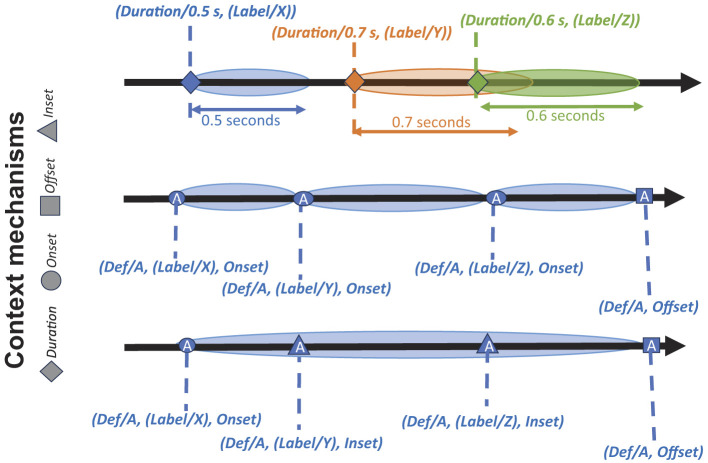
Basic HED event process representations. Each thick black arrow represents an experimental timeline illustrating a way of representing events in the experiment data. Extended colored ovals represent event processes. HED event-context tools associate *event phase markers* (diamonds, circles, etc.) with all available information concerning the associated event process as “context.” **Top timeline:** Three colored ovals represent three distinct event types. Here, HED *Duration* tags are used to implicitly mark event processes. The *Duration* tags appear at the event markers of the event onsets (marked by diamonds) and the offsets are calculated by adding the *Duration* values to the onset times. Three unrelated event processes are shown—each begins at an *Onset event phase marker* (diamond) and is associated with a *Duration* tag group rather than an explicit *Offset* marker tag. Note that here the orange and green event processes overlap in time. The **Middle timeline** illustrates the use of event *Onset* marker tags for a sequence of three event processes (blue ovals) of the same type that are thereby assumed to be temporally contiguous. Here event type information is given as *Def/A* using the HED *Definition* mechanism, with individual modifiers *Label/X, Label/Y*, and *Label/Z*, respectively. In BIDS-formatted datasets, HED definitions are stored in files with names ending in *_events.json*. This same timeline might also be annotated as a single encompassing *Def/A* type event with two *Inset* phase markers, as shown in the **Bottom timeline** annotation. Here, two *Inset* tags mark internal *event phase transitions* (for example, the event here might be a movie scene realized as a sequence of three camera shots, or a reaching movement with two observed course corrections).

HED currently has robust representation mechanisms for supporting most types of context-related search and analysis as illustrated in Figure 2. The top timeline illustrates the *Duration* tag for representing event processes. The tag (*Duration/0.5 s*, (*Label/X*)) indicates an event that starts at the time marker associated with this expression and ends 0.5 seconds later. The *Label/X* is a placeholder for any group of HED tags describing this event. Events annotated using *Duration* can overlap in any way and each group is a separate event process.

The middle timeline of Figure 2 illustrates annotating a series of three temporally contiguous event processes of the same type using explicit HED *Onset* and *Offset* tags. *Onset* and *Offset* tags must be grouped with a *Def* tag representing a named group of tags defined elsewhere by a *Definition* tag. The first event process (*Def/A*, (*Label/X*), *Onset*) begins at the indicated time marker and is described by the tags associated with *Definition/A* as well as the additional tags given in the *Onse*t group (in this case just *Label/X*). These event processes begin with an *Onset* group and end either with another *Onset* group of the same type (the second oval on this timeline) or by an *Offset* group of the same type (the last oval on the middle timeline). Using *Def* to anchor the annotation allows to match *Offset* tags at a later time-marker to the appropriate *Onset* marker, enabling the representation of virtually any type of overlapping event process configuration. Definitions of the form (*Definition/A/#*, …) allow users to generate a family of definitions where values from a column such as ID column in an events table might be substituted for the # to reference individual event processes within the family.

### 3.3 Practical context handling

**Figure 3:**
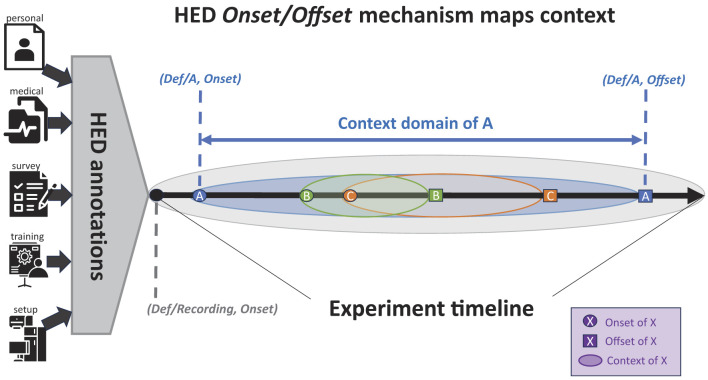
Representation of event context for a data recording. Here, two temporally overlapping events (B and C) occur during event process A. All events occur within the encompassing *Recording* event. See text for details (icons from https://www.thenounproject.com).

We now look at how the five types of event context described in Section 2 may be derived from HED event annotations. Automated event context representation requires the standardized tag vocabulary and syntax HED provides, as well as useful context representation mechanisms. Ongoing event context is straightforwardly represented using the mechanisms of Section 3.2. Users typically define frequently occurring event types using HED definitions (usually only once for the dataset in a top-level *events.json* file). Once defined, instances of these event processes can be designated in the event file using event markers associated with *Onset* and *Offset* tags (or alternatively *Duration* tags). Priming and environmental context information can be captured by a single recording event process that begins at the beginning of the recording and offsets at its end, as illustrated in Figure 3 by the encompassing gray oval.

Figure 3 shows an encompassing *Recording* event process (here, the event process shown in Figure 3 as a gray oval) whose *Onset* is the beginning of the recording and whose *Offset* is its end. This HED convention is used to gather, store, and make available annotations that are in effect during the entire recording (including information concerning the participant, recording environment, any pre-recording participant priming, and parameters for the recording itself). It can also serve as a place to store *Note* tag text entries concerning unplanned circumstances affecting data quality, etc.

An annotator may insert and populate an explicit *Def/Recording* event on setting at time zero in the event table (a filename ending *_events.tsv* in BIDS). However, the HED tools do not require this as HED tools can gather HED annotation-relevant *Recording* information stored in other files in BIDS-formatted datasets. For example, under BIDS each experiment participant is required to have an entry in the *participants.tsv* file giving associated participant metadata. BIDS recommends that age, sex, and handedness be included in this top-level *participants.tsv* file and accepts the designations *left, l, L, Left*, or *LEFT* in the handedness column to indicate that the participant is left-handed. In all these cases, these tags can be unambiguously resolved by the HED tag *Left-handed* for annotations in the *participants.json* file. Other information, including an added HED column, may be optionally included. Similarly BIDS *scans.tsv* files can be used to contain information about parameters used in recording the data. When present, HED tools can incorporate this information into a *Recording* event and the ongoing context. The challenge for HED here is to know where the relevant metadata are stored and how they are represented.

### 3.4 Other types of context representations

In this section we discuss context information that cannot yet be directly represented in HED and potential paths to incorporating this information into data analysis workflows.

#### 3.4.1 Unstructured data

Unfortunately, there is an enormous range of other unstructured, but potentially relevant information concerning events and their experience whose descriptions HED has not yet standardized. To make use of any available non-standardized *participant context* information, text descriptions (even excerpts or data from papers, organized in a specified manner) might in future be digested by *text feature processors* to produce a limited number of standardized factors. The feature processors might include encodings generated by large language models (LLMs) (Mitchell and Krakauer, 2023), by latent factor analysis (Cooper et al., 2019), or by other feature extraction techniques. Once extracted in some standardized form, these features could be used downstream, such as in analyses seeking to parsimoniously explain variance, to identify independent information sources, or to look for commonalities among subjects or among brain measures or source activities. Feature processor tools should have standardized inputs and outputs so that standardized analysis tool sets could use them downstream in analysis.

#### 3.4.2 Preceding event context

**Figure 4:**
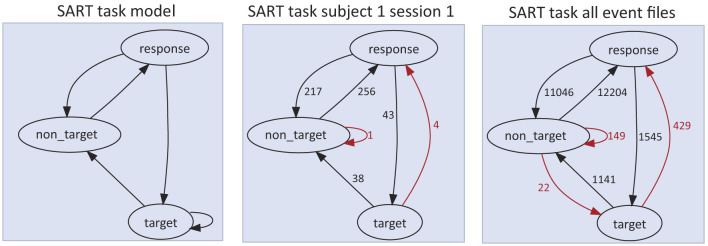
Illustration of HED tools produced event transition count maps for event files of dataset *ds004350* (available for inspection, download or high-performance computing at https://www.nemar.org/dataexplorer/local?search=ds004350). In SART (Sustained Attention Response Task), the participant is shown a succession of digit images (“0” through “9”) in random order and told to press the spacebar immediately after the appearance of any digit except the target digit (here, “3”). In each directed graph, nodes represent event markers and an edge *A* → *B* means that the event marker A is followed by event marker B. The counts on the edges represent the number of times the event transition appears in the event data. Red arrows designate event transitions inconsistent with task instructions. **Left panel:** Event transition graph for a session in which the participant made no errors (i.e., followed task instructions throughout). **Middle panel:** Task events for Subject 1/Session 1. Four *target* (“*3*”) presentations were followed by false alarm *response* events, and one *non_target* incorrectly by a miss (not a *response* event). **Right panel:** The consolidated sequence graph for all 46 compatible SART event files including in total 26,582 events; of these, 600 event transitions (2.3%) contravene task instructions.

Analysis of the effects of preceding event context is by its nature, task and experiment specific. Typically, when study of relationships between successive events is a research goal, the analyst must write code to identify events for which a relevant sequence of previous events has occurred. Simple event transition statistics can provide valuable statistical information to either infer task expectations (and thus, participant intentions), or (when the task expectations are known) to interpret vagaries of actual participant behavior. Figure 4 shows (on the left) the SART task design as an event type transition network in OpenNeuro/NEMAR dataset *ds004350*. The actual event transition counts for one participant session and across all participant sessions are shown in the middle and right panels respectively.

The event transition count diagrams shown in Figure 4 provide a clear overview of participant behavioral patterns in the experiment, though their interpretation relies on separate knowledge of the task paradigm. Supplementary Figures 5, 6 show event transition graphs for the other tasks performed by participants in the same dataset sessions. Event transition graphs have potential uses in experiment visualization, task representation, context enhancement, and verification. Context provided by more than one preceding event can affect behavior and brain dynamics; to more fully explore such possibilities, more abstract analysis and visualization methods must be used.

#### 3.4.3 Spatial context

Currently, HED vocabulary supports only rudimentary means of specifying *spatial relationships* between the participant and sensory presentations, between sensory elements and/or environmental constraints, and in describing motor actions and their spatial relationships to environment, objects within it (e.g., the computer screen), and to the actions of others in social neuroscience experiments or in 3D virtual reality paradigms.

Further, in a neuroimaging experiment involving movie viewing, a participant may be sitting in a chair viewing a display screen (or a virtual screen in a heads-up display) depicting a 3D environment within which actors, animals, and objects move. Participants usually follow movie action by, as it were, virtually projecting themselves into the 3D scene, translating the 2D moving images into movement within the visually suggested 3D environment.

A fully annotated experiment dataset of this type might capture spatial relationships between the participant, screen, and imaged world, however HED does not yet include syntax, vocabulary, or tools for doing this. Nor does HED yet include adequate standardized support for describing events in mobile brain/body imaging (MoBI) experiments that combine EEG and body (and/or eye) movement recording of participants moving naturally in 3D space.

#### 3.4.4 Imperative event context

A great challenge for HED development remains the handling of *event imperative context* that is most typically created by participants following (or not) task instructions (simple example: “Press button B following each presentation of stimulus A”). Adding syntax to the HED specification to express event imperatives is important, as motivation or *intent* is an essential aspect of action. A fundamental problem for cognitive neuroscience is to understand how our brain activity supports motivated (as well as inadvertent) actions in different contexts, since this is fundamental to its central goal to understand how brain activity supports human experience and behavior. To retain and convey information concerning relationships between events and in particular, sensory presentation events and motor actions), HED needs to encode task event relationships in a standardized way. Statistical event transition graphs for a dataset (Figure 4) may be helpful in this regard, but inferring intention and task directions from statistics is in general difficult and prone to error.

Consider the SART task of Figure 4. Here, presentation of any but the target numeral initiates both a *visual stimulus presentation event* (process) and begins a coincident *action imperative* that ends when the participant presses within or else withholds a key press throughout some task-imposed or analysis-specific post-stimulus period.

Typically, researchers record the degree of success of task action imperatives by coding *post hoc* the task-related significance of participant responses, e.g., scoring participant responses (most simply, as correct or incorrect) in relation to one or more task action imperatives in one or more custom columns in the event log for the data recording. Simply recording response scores in this way (e.g., within a supplementary column in BIDS *events.tsv* files) does not capture the nature of the task action imperative itself, information that may be important in further analyses and/or in cross-study analyses run that include data from this experiment.

It also requires the user or data analyst to build custom analysis scripts to populate and/or interpret these column values for each dataset. Building HED tools to generate such information columns more automatically from a formal task description would further ease both the HED annotation process and the creation of HED-supported data analysis pipelines. This alone discourages, at the least, development of analysis pipelines operating across available (published or still private) neuroimaging datasets, no matter how appealing the prospects of such analysis might now appear.

Therefore, we would like to employ (and contribute to developing) a *standardized task representation language* that can be incorporated into HED data annotations. The resulting task representations could be used to build experiment control scripts. They could also be used by HED to add information concerning *event imperatives* to annotations of both action and sensory events. They could allow construction of real time performance monitoring and HED annotation during neuroimaging sessions. This would greatly reduce the *post hoc* event annotation burden on experimenters, ensuring more complete recording of critical information within recorded datasets.

## 4 Discussion

### 4.1 Machine-actionable data with HED

The HED project aims to provide standardized vocabularies and software tools whose use together justifies the expenditure of effort to record detailed information about experiment events in the form of HED strings to increase the possible scope and depth of further analysis of the data itself and/or considered in concert with other available datasets. It should be emphasized that while HED can provide useful summaries and visualizations within datasets and across datasets, its primary mission is to support annotation, queries, and analyses at the recording and event levels. While the linked data representations provided by ontologies are supported by a robust query infrastructure, their usage is generally confined to high-level exploration at the dataset level. HED provides more specialized fine-grained searching mechanisms for event-related analyses. Further, recent tool development in the HED ecosystem focuses on providing better pathways for integrating information from other sources of both structured and unstructured information.

We and the HED Working Group have attempted over the last 4 years to advance the HED specification and software framework to the point where it has the mechanisms to effectively handle several types of contexts in a machine-actionable way, so that datasets can be combined for analysis without extensive code development and information re-coding. Significant progress has been made in the effective representation of temporally extended events on the experimental timeline and their combination with context information for analysis. HED tools have extensive search and summary tools as well as tools to extract relevant data epochs and design variables from HED-encoded event information.

However:

1. It may be infeasible to exhaustively incorporate *all* available and potentially relevant context information in a standardized manner.
2. Standardization of the possible types of context information will likely occur incrementally. As data standards evolve, more of this information will be standardized. Even when terminology for one type of context data is standardized, all older and many newer datasets will not include this type of data in standardized form.
3. The potential size and diversity of context information requires custom filtering suited to each proposed analysis. While it is not possible to pre-specify all possible useful context filter types, their machine and user interfaces should be standardized to allow new filters to be built and used efficiently.

Context filtering functions will also need to have wrappers that read the standardized output of other context filters and, after the information is digested, output it in a standardized manner so that analysis tools exploiting the output can be developed using a standardized API. We have made some progress on the HED-related aspects of this framework for *preceding event context*, in building HED remodeling tools that provide a limited number of operations for detecting event sequences based on HED tagged event codes.

#### 4.1.1 Is text-based annotation necessary?

While building out data representation systems including the HED syntax, terminology, and tools, we might consider in what ways and cases is text-based event representation or mediation optimal or in some cases necessary, given the impressive demonstrations of the abilities of new AI models to combine data in whatever formats it is presented to mimic its interrelationships. Training such models is, however, inherently expensive, and accuracy of their resulting models is yet far from guaranteed. It is not clear whether or how these models can be dissected to discover the lower-dimensional structure in the data they may be exploiting, though mathematical data science research in this direction is intensifying. It seems possible that training networks on diverse, synchronously recorded data streams, for example brain activity plus body and eye movements, likely augmented with text-based annotations, might in future prove of both use and scientific interest.

### 4.2 What is worth annotating? For whom?

Annotating data in an *analysis-ready* form for archiving and/or sharing requires effort whose extent depends on the quality of annotation software tools available, and the depth and breadth of specificity of the annotation. Attitudes do also matter: one of us can recall a mentor, now over 40 years ago, abruptly telling an inquiring caller to, “Get your own damn data!” Today, there is more general agreement that carefully recorded data can be and should when possible be treated as an invaluable scientific resource—though understanding is still less common that to be truly of further use, the data must be adequately annotated (i.e., thereby remaining not just as obscure or meaningless “piles of numbers” occupying space in some repository, but shared in truly “analysis-ready” form). Research funding agencies are also gradually raising requirements for sharing data whose acquisition they fund, while supporting construction of data archives and neuroinformatic standards to support effective data sharing and reuse. But an ever-important factor remains the philosophy and goals of data authors: Do they believe that there is information in their data that can and should be mined, likely using techniques other than those they themselves have applied to it? And, how may they themselves benefit from making it possible for themselves or others to do so?

Because of the relatively low numbers of participants and/or recording times in most neuroimaging studies, the adoption of analyses using a single, relatively low-information value measure (e.g., ERP/ERF peak amplitude condition contrast), the relatively low statistical power of planned event-related data measure contrasts prevents interpretation of observed differences in many dimensions of stimulus, stimulus sequence, and/or task condition variation. Unfortunately, this has encouraged researchers to record and report only minimal event information required for their planned basic analyses, rather than annotating the data event record with an eye to its possible future uses for other purposes including across-study analyses whose larger size could make feasible the statistical interpretation of more event-related contrasts.

#### 4.2.1 What can be annotated?

The factors that influence brain responses and human behavior are myriad, and many not practically measurable. Finite neuroscience experiments can only control and record in detail a limited number of informative dimensions. Yet, uncovering generalizable factors binding brain dynamics, experience, and behavior and their interrelationships—may be essential to true understanding, principled assessment, and confident prediction. The potential benefits of analyzing collections of diverse experiments to mitigate this lack of knowledge are clear, as are obstacles to this approach.

We believe that a most practical approach to unraveling the complex influences of context on brain dynamics supporting cognition and behavior in neuroimaging experiments is a hybrid/evolutionary approach. For information that has not been (and may not soon be) standardized, this would provide as much detailed information as possible within text blocks labeled as belonging to broad, inexactly standardized categories. For personal context, for instance, these categories might be *demographic, medical, genetic, physical, behavioral, social*, and *current state*.

#### 4.2.2 Two research directions

Today we see two movements advancing within the neuroimaging community. One, mounted in response to critiques of many reports in the literature (Kriegeskorte et al., 2009), seeks to minimize the risk of reaching and reporting unsupportable conclusions reached through poor statistical practice or interpretation. A resulting movement asks data authors to formally register the hypotheses, tests, and test measures they intend to apply to their data before the data are collected (Poldrack et al., 2017). This point of view prizes data uniformity and reproducibility; annotating event details not entering directly into the planned analysis is at best peripheral to the experiment goals, or even (in its most extreme form) to be discouraged. Certainly, if reproducibility is the ultimate goal, the incentive to annotate the data sufficiently to support further analysis is at best minimal.

A complementary movement, currently surging in neuroimaging, eagerly seeks large collections of hopefully well-annotated basic research and/or clinical data incorporating wide variability in task conditions, stimulus details, etc. This movement aims to apply new, highly complex machine learning methods to rich collections of varied data, hoping thereby to build networks delivering reliable and useful biomarkers when applied to a wide range of real-world data (Li et al., 2019). From this point of view, recording details of events occurring during the neuroimaging experiments is a potential boon to potential discovery and application development, as the trained networks that learn to measure a desired biomarker also learn to actively ignore data variability, potentially enabling unforeseen discoveries concerning aspects of the model structure other than those directly producing the intended biomarkers. The need for well-annotated data is now widely recognized in the field of AI development, learning to broad efforts to coordinate development of annotated datasets for a wide range of purposes (Thompson et al., 2020; Valdes-Sosa, 2021; Budin-Ljøsne et al., 2023).

Regardless of viewpoint and direction, detailed annotations of all aspects of the experiment, participant, and analysis process are invaluable in determining both sources of bias and potential contrasts for fine-grained analysis of effects. Unfortunately, however, relatively few active science laboratory principal investigators are now well equipped to undertake research in these new directions with their students and associates, and thus may have little initial enthusiasm for marshaling the lab resources in time and skill learning needed to make their shared data analysis ready for a range of purposes. As thousands of eager students come out of new data science programs being mounted at leading universities, the diffusion of new data science and AI methods into cognitive neuroscience will thus be a prolonged process. Indeed, one might term it a scientific culture change. Unfortunately, in the meantime, decades of carefully designed and acquired human brain/behavioral experiment data resting on disk drives may not be shared in a form that is truly analysis-ready. The HED Working Group is thus eager to take advantage of any opportunities to inform researchers of the potential value of adding HED descriptions of experiment events to their data.

### 4.3 Ongoing challenges

Historically, much task-based analysis of human electrophysiological experiment data has focused on dynamic measures of the recorded electromagnetic brain dynamics during time periods time-locked to phase markers of similar (and formally, considered equivalent) events. Annotation of *events in context* in all these designs poses several ongoing challenges we and the HED Working Group are working to address:

- Building tools to construct event context including *priming* and *preceding event context* information. As this can be voluminous, these tools should take user input as to the prevailing context information that is of interest for the analysis.
- Building notation, syntax, and tools to annotate relationships of task mediated relationships between one or more events and subsequent events (e.g., *action imperatives* to produce a subsequent action in response to one or more task events).
- Building notation, syntax, and tools to annotate, more generally, task-directed (or other) intentions of participants. This requires developing a *representation of participant task(s)*. The HED sequence graphs (Figure 4) are one start in this direction. A possible approach is to build a somewhat abstract, special purpose task specification language for which translators of experiment scripts written for common experiment control applications might be developed.
- Building HED libraries of terms to describe *spatial representations* (e.g., 2D screen coordinates, 3D scenes on 2D screens, 3D experiment space coordinates, and 3D peripersonal coordinates). Augmented and virtual reality environments offer additional challenges to creating annotation methods and notation that are simple enough to invite regular use. The increasing availability of in-phone 3D scanners and scanning software built using AI modeling may provide a practical pathway for introducing these representations into the data.
- Building workable spatial representations of participant and other agent *body positions, poses, and movements*. This effort will need to combine expertise in animation and in biomechanics. Again, AI-based methods for modeling body position, pose, and movements directly from video are rapidly progressing.
- Developing meaningful representations of event details in several important areas requiring concerted development within the respective research communities. The HED Working Group is now contributing to a HED library schema for language terms and is considering how to create a term library for video. Schemas for music, for other arts, for interpersonal interactions, and other topics could add considerably to the wealth of available analysis-ready neuroimaging data for new analysis methods to explore.
- Extending HED to combine session, participant, and session event annotations with world knowledge bases, particularly neuroimaging atlases, both anatomic and brain dynamic. Such a facility could both simplify and intensify exploration of how brain dynamics support human experience and action, both in health and pathology.

Underlying the need to broaden the scope of HED annotation is the need to identify suitable vocabularies for particular research fields and subfields. Creating new HED library schemas by “HED-ifying” already well-defined vocabularies (assuring that the terms are unique, clearly defined, and stand on their own, and identifying hierarchical relationships and attachment points in the standard HED schema) can be relatively straightforward. However, persuading researchers in a specialized field to agree on terminology can be the bane of any standardization effort. Here then, proceeding by combining first mover advantage with crowd-adoption testing is perhaps the only effective way to go forward.

One of the greatest challenges of standardization to enable effective advanced and across-studies analysis is that researchers usually only annotate events relevant to the analysis they envisioned when conceiving the experiment—not all the event information that might become event data and/or context needed by further analyses. Researchers are often reluctant to “clutter” the data record with more information than needed for their own analysis purposes. Good, user-friendly context filtering mechanisms could at least lessen this issue by assuring that tools could always deliver cleanly filtered data annotations, stripped of any information of no interest to their immediate analysis goals.

### 4.4 Experimental data as the legacy

We predict a cultural shift toward regarding the data and metadata produced by and involved in creating and running an experiment as an important, if not the most important experimental research product.

Certainly, the data and metadata, when effectively stored and shared, are now becoming the longest-lasting legacy of experimental neuroimaging research. Our goal is to make the HED event annotation system tools and infrastructure ready to support a shift to sharing, to the maximum extent possible, neuroimaging data in a form supporting and rewarding exploration using new tools and methods by both current and future scientists.

Publishers now encourage authors to submit details of their shared data in a separate, citable publication. More effort must be expended to realize and optimize the value of shared data, and its citations—to both data authors and future data users. A collaborator recently recounted to us their request to a funding agency to extend a relatively large study in which they were collecting *and* immediately sharing the collected data. They asked the agency to compare the number of scientific papers written on the data collected in other supported projects (a few) against the by-then impressive number of papers that had so far been written on the project data they had collected and shared.

Both granting agencies and academic advancement committees must come to recognize the value to science and society of well-acquired and annotated data, collected in well-designed paradigms, and shared *in analysis-ready form* by experimental researchers. They must similarly recognize the value of computationally oriented researchers who make use of shared (or, combined original and shared) data to advance scientific understanding or its applications, and weigh the contributions of both data contributors and data users appropriately in the career advancement process.

In conclusion, the current and ongoing need and opportunity appears bright for more optimal and widely known and applied text-based event annotation to accelerate progress in neuroimaging research in the coming decade and beyond, though continued development, dissemination, and widespread recognition and adoption of best annotation practices will require considerable, widespread, and dedicated effort. In large part spurred by the widespread recognition of the power of new analysis approaches applied to well-annotated data, event annotation is now increasingly considered important and timely by neuroinformatics experts and increasingly, by neuroscience leaders. We in turn hope that event annotation of neuroimaging data can contribute to the development of new, more complete and detailed models of brain dynamics supporting experience and behavior in health and disease and are building the Hierarchical Event Descriptors (HED) system to support this process. We invite others interested to join us (https://www.hedtags.org).

## Data availability statement

The original contributions presented in the study are included in the article/Supplementary material, further inquiries can be directed to the corresponding authors.

## Author contributions

SM: Writing – original draft, Writing – review & editing. KR: Writing – original draft, Writing – review & editing.
