# Supplementary material for: Events in context—The HED framework for the study of brain, experience and behavior
Source: Front Neuroinform. 2024 May 23;18:1292667. doi: 10.3389/fninf.2024.1292667 (PMC11153828; doi:10.3389/fninf.2024.1292667)
Supplement: Supplementary file 1 [file Data_Sheet_1.pdf]

Tables

1. Supplementary Table 1: Primary URLs for tools and tutorials.
2. Supplementary Table 2: Example HED search queries.

Figures

1. Supplementary Figure 1: Examples of HED design matrix extraction.
2. Supplementary Figure 2: Template extraction.
3. Supplementary Figure 3: Remodeling transformations for summary and visualization.
4. Supplementary Figure 4: HED in BIDS.
5. Supplementary Figure 5: Graph of the LG (local-global) dataset.
6. Supplementary Figure 6: Graph of the N-Back dataset.

Table 1: Primary links to HED-related resources

URL	Description
https://www.hed-resources.org	Central site for all HED documentation
https://www.hedtags.org	HED homepage
https://www.hedtags.org/display_hed.html	Central viewer for all vocabularies
https://github.com/hed-standard	HED GitHub organization housing all source
https://hedtools.org	HED online tools
https://hed-specification.readthedocs.io/en/latest/	HED specification document
https://github.com/hed-standard/hed-specification/tree/master/tests/json_tests	JSON unit tests linked to specification

Table 2. Simple HED tag queries. HED provides a robust query mechanism including the simple tag queries shown in this table. In addition to simple tag search, HED also supports more complex queries that include logical expressions (e.g., and, or, not) and queries detecting tags that are grouped together (e.g., *[Red, Triangle]* only returns true if the expression has a parenthesized group containing both *Red* and *Triangle*). From https://www.hed-resources.org/en/latest/HedSearchGuide.html for more examples.

Query type	Example	Match	No match
Single-term<br>Match the term or any child. Don't consider values or extensions when matching.	*Agent-trait*	*Agent-trait*<br>*Age*<br>*Age/35*<br>*Right-handed*<br>*Agent-trait/Glasses*<br>*Agent-property/Agent-trait*<br>*(Age, Blue)*	*Agent-property*
Quoted-tag<br>Match the exact tag with extension or value	"*Age*"	*Age*<br>*Agent-trait/Age*	*Age/35*
	"*Age/34*"	*Age/34*<br>*Agent-trait/Age/34*	*Age/35*
Tag-path with slash<br>Match the exact tag with extension or value	*Age/34*	*Age/34*	*Age*<br>*Age/35*<br>*Agent-trait/Age/34*
Tag-prefix with wildcard<br>Match the starting portion of a tag and possibly its value or extension.	*Age/3**	*Age/34*<br>*Age/3*<br>*Agent-trait/Age/34*	*Age*<br>*Age/40*

(A) BIDS *events.tsv* file + sidecar

onset	duration	event-type	HouseFactor	FaceFactor
0.203	n/a	h	1	0
1.234	n/a	h	1	0
4.603	n/a	f	0	1
6.011	n/a	f	0	1
7.302	n/a	h	1	0

```
{
  event_type: {
     HED: {
        "h": "Condition-variable/House, ...",
        "f": "Condition-variable/Face, ..."
     }
  }
}
```

(B) BIDS *events.tsv* file with HED column

onset	duration	HED	HouseFactor	FaceFactor
0.203	n/a	*Condition-variable/House, ...*	1	0
1.234	n/a	*Condition-variable/House, ...*	1	0
4.603	n/a	*Condition-variable/Face, ...*	0	1
6.011	n/a	*Condition-variable/Face, ...*	0	1
7.302	n/a	*Condition-variable/House, ...*	1	0

(C) BIDS *events.tsv* file with and without *Onset*

onset	duration	HED	LeftFactor
0	n/a	*(Def/LeftSym, Onset), ...*	1
0.203	n/a	*Sensory-event, ...*	1
1.234	n/a	*Agent-action, ...*	1
4.603	n/a	...	1
6.011	n/a	...	1
7.302	n/a	...	1

onset	duration	HED	LeftFactor
0	n/a	*Def/LeftSym, ...*	1
0.203	n/a	*Sensory-event, ...*	0
1.234	n/a	*Agent-action, ...*	0
4.603	n/a	...	0
6.011	n/a	...	0

*(Definition/**LeftSym**, (Condition-variable/Left, ...))*

Supplementary Figure 1: Design matrices and factor matrices in HED. HED can extract factor matrices for any supported HED query (see Supplementary Table 2), but the *Condition-variable* tag is particularly important because it conveys the annotator's intent that this is part of the experimental design. Three dots (...) means that any number of other HED tags may be included in the indicated HED string. **(A)** An example of a BIDS event file with a categorical column named event-type. The values in this column (h or f) are annotated in an accompanying JSON sidecar (to the right of the table). HEDtools can produce factor columns to indicate the experiment design. Here 1 indicates that the factor is present and 0 indicates that it is absent. **(B)** Same as A, except that the HED annotations are included directly in a HED column in the *events.tsv* file. (C) Definitions can be used with HED context mapping and *Onset* and *Offset* to annotate an ongoing condition. In the left table, the first event indicates that the *Def/LeftSym* condition starts. Since there is no *Offset*, the condition continues, and the factor vector is all 1's. Compare with the right table where the *Onset* is not used.

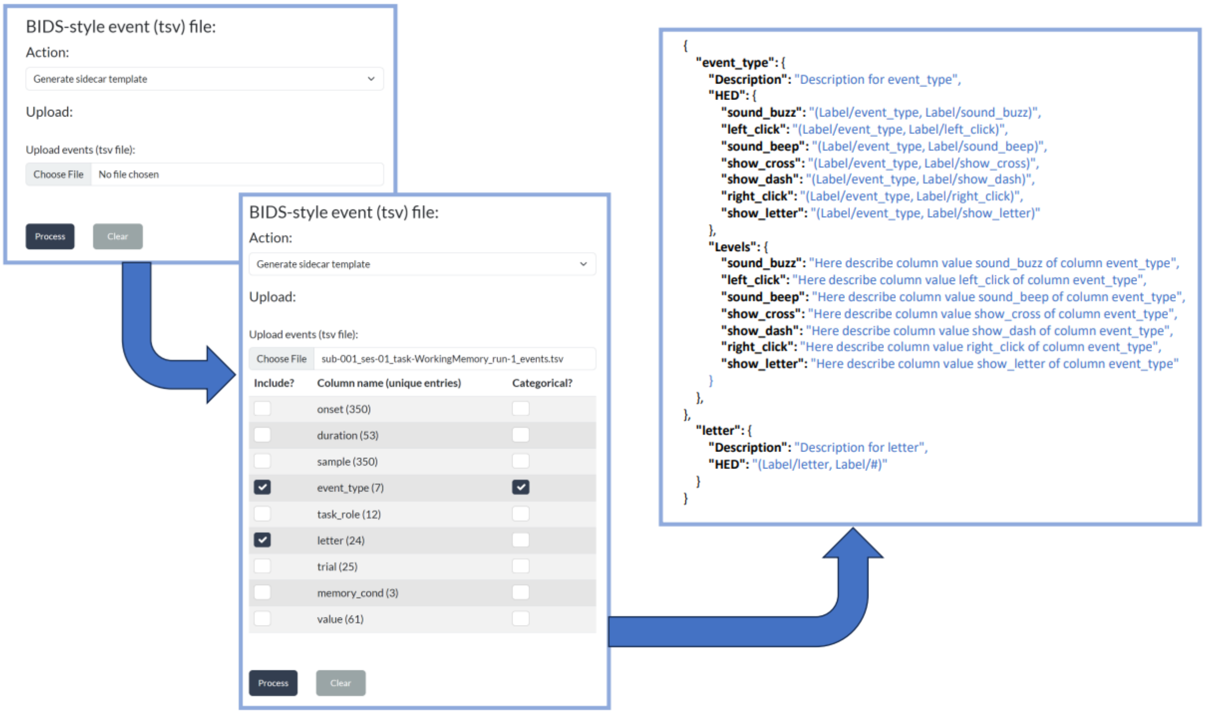

Supplementary Figure 2. Extraction of a template JSON sidecar using HED online tools. The process of generating a JSON sidecar template from a BIDS event file. **Left panel.** The “Generate sidecar template” action has been selected from the pull-down. After selecting a BIDS event file using the file chooser and pressing Process, the middle panel appears. **Middle panel.** The online tools now display the number of unique values in each column of the selected file *sub_001-ses-01_task_WorkingMemory_run-1_events.tsv*. The user must select which columns to include in the template (left column of checkboxes) and which of the included columns are to be treated as categorical columns. In this case the columns *event_type* and *letter* are included and event_type has been selected as a categorical column and letter defaults to a value column. Categorical columns have annotations for each unique value in the column while value columns just have a single annotation for the entire column. **Right panel.** The resulting JSON sidecar template produced after the user presses *Process*.

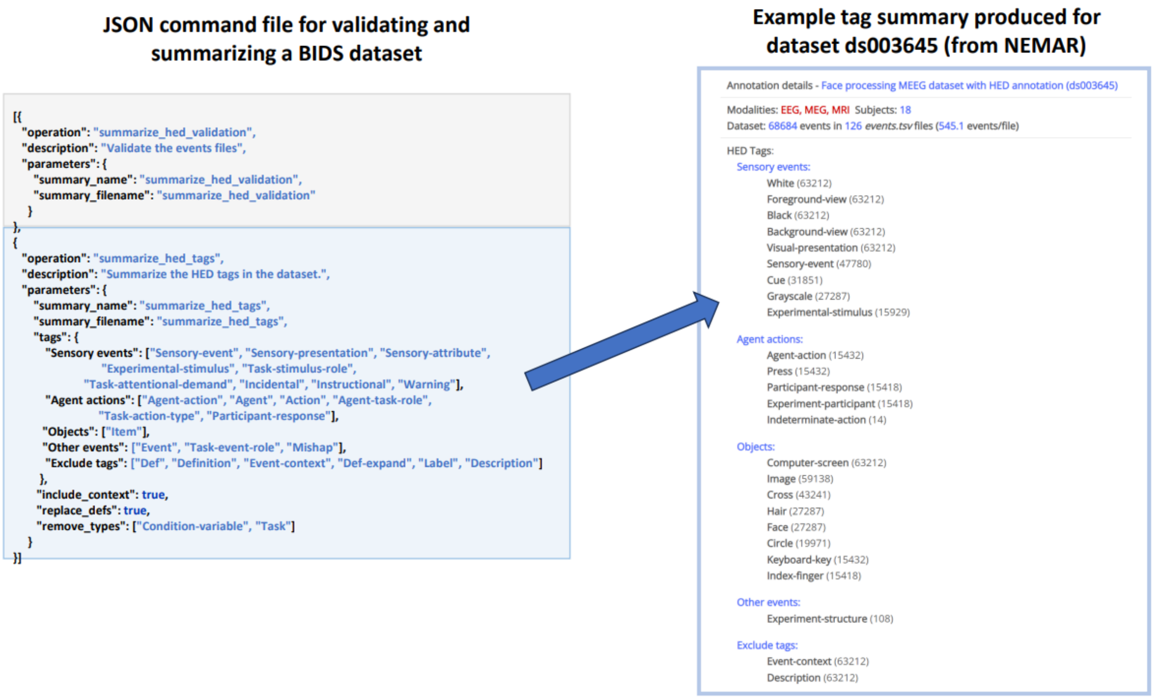

Supplementary Figure 3. HED remodeling tool suite for summarization. The remodeling tools allow users to specify and execute transformations and other operations on *.tsv* files without coding. The operations are specified as a list of text commands in a JSON file. HEDTools are available to execute the operations on a BIDS dataset or on any directory tree. The command file can have any number of operations listed. **Left panel:** an example command text file with two operations: validate (gray box) and summarize the HED annotations (light blue box). All operations require an operation name and a description followed by an operation-specific dictionary of parameters. Transformation operations transform the input *.tsv* files into output *.tsv* files. Both the validation and the tag summary are examples of summary operations, which produce output data files rather than transforming the input *.tsv* file. The validation summary (*summarize_hed_validation*) produces a file of validation errors (if any), while the tag summary (*summarize_hed_tags*) produces summaries of the HED tags used in the annotation. **Right panel:** Output of the *summarize_hed_tags* command for dataset ds003645 as displayed on NEMAR (https://nemar.org). Notice that the tags dictionary in the command specifies how the output should be organized. The keys of the tags dictionary correspond to the blue titles. Under the section "Sensory events", the tags *White*, *Foreground-view*, *Black*, *Background-view*, and *Grayscale* appear because the tags dictionary specified that *Sensory-attribute* tags should appear in the "Sensory events category" (blue arrow).

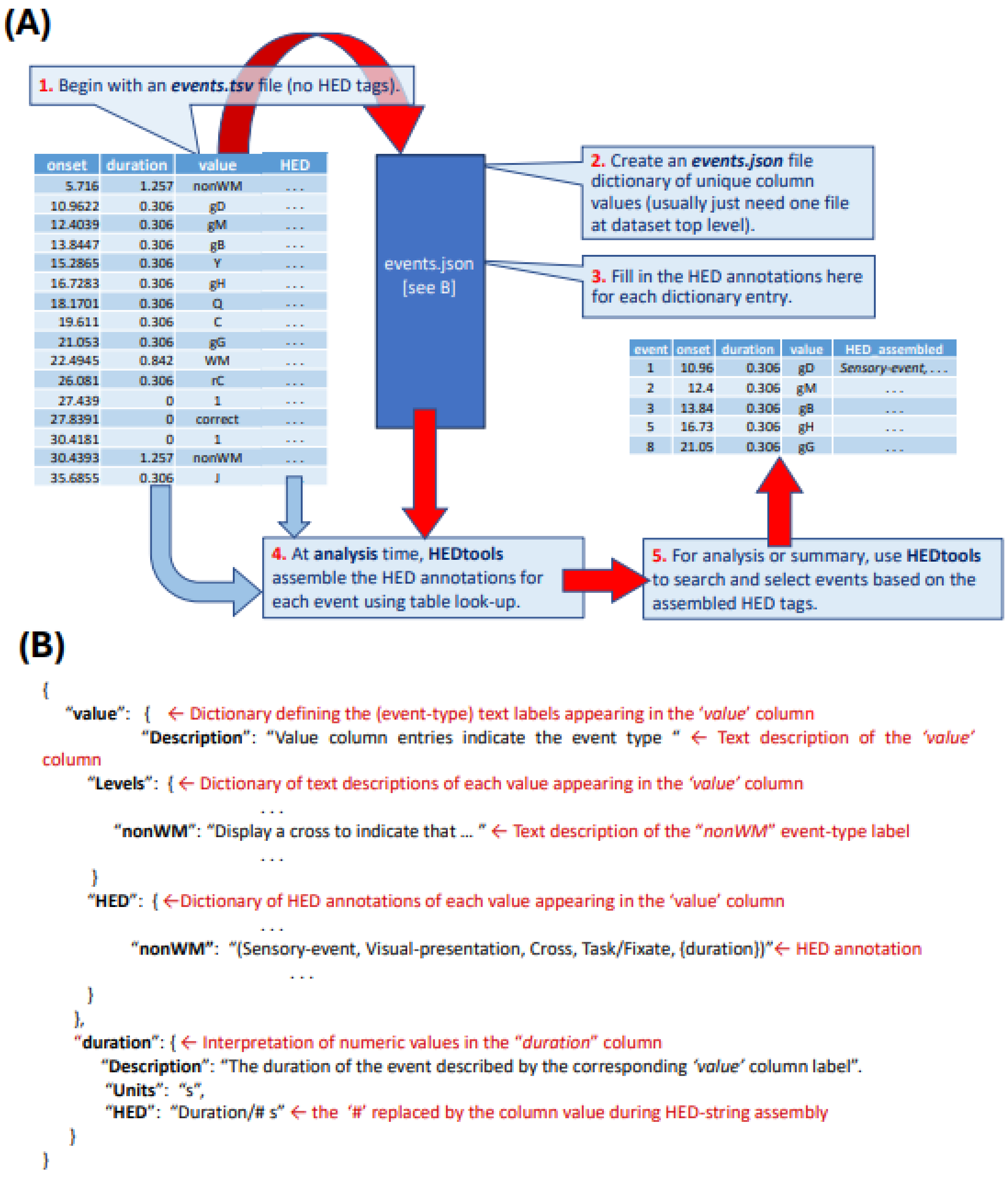

Supplementary Figure 4: The annotation/analysis process in BIDS: **(A)** The overall flow and steps in using HED in BIDS. (1) BIDS stores event marker information for a recording in rows of an *events.tsv* files. Here, stimulus presentation and button press response event codes output by the experiment control program are listed in the *value* column. While it is possible to post HED tags describing each event in a HED column in the same file, a more efficient approach (2) uses a dictionary of *.tsv* file column names in an associated *events.json* file (excerpted here in B). When the same labels (3) are used to describe events in each dataset session, this *.json* file can be a single file at the top level of the dataset file hierarchy. Then, to perform a requested

event search and selection, (4) the full HED string assembly tool assembles a complete HED annotation for each event, and (5) passes the assembled HED string (augmented with event context information) to HED event search tools. **(B)** Excerpts of the accompanying *.json* sidecar file dictionary for the label (aka *'Level'*) and value entries in the *.tsv* file. Note: the {duration} annotation that appears in the HED definition for **nonWM.** The contents of the curly brace must be a column name. The curly brace indicates that the HED annotation for that column is substituted for the curly braces. Thus, when the HED annotation for the first row of the event file in (A) is assembled, the annotation will be *(Sensory-event, Visual-presentation, Cross, Task/Fixate, Duration/1.257 s)*, indicating that the the event represents the presentation of a fixation cross for 1.257 s.

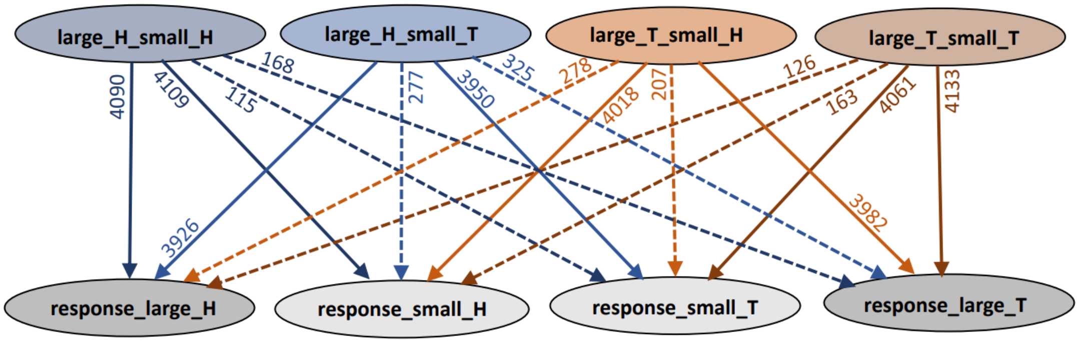

Supplementary Figure 5: Graph of the LG (local-global) task from dataset ds004350 from openNeuro. In this task participants were presented with one of 4 images each consisting of a large outline letter filled with small letters. Only the letters 'H' and 'T' were used. Two of the images were congruent (large 'H' filled with small 'H' and large 'T' filled with small 'T'), the other two were incongruent (large 'H' filled with small 'T' and large 'T' filled with small 'H'). On each trial the participants were asked to respond to the large letter (indicated by "response_large…") or the small letters (indicated by "response_small…"). In the following graph, the nodes indicate values in the "value" column of the event files for the dataset. The directed edges indicate the responses directly following the image presentations. Dotted edges indicate incorrect responses. The graph shows that participants made significantly fewer errors when the images were congruent.

Notes on data: The dataset had 48 event files containing a total of 72,223 events. In the event files "large_H_small_" was incorrectly coded as "ignore". The following files were not included due to using a different encoding of events: *sub-4_ses-2*, *sub-5_ses-1*, and *sub-5_ses-2*. **Notes on the graph:** The back edges from responses to images were not displayed as they indicate transition to the next trial. Also, there were 22 image-to-image edges (not displayed) indicating trials in which the participant did not respond.

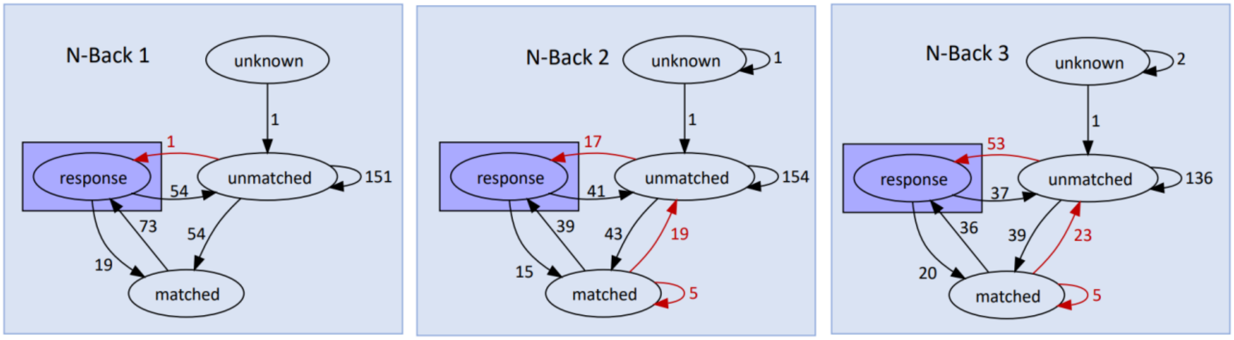

Supplementary Figure 6: Graph of the *N*-Back recordings for sub-1_ses-1 of ds004350 on openNeuro (*https://openneuro.org*). The experiment used a standard *N*-Back paradigm – images containing single letters (one of "A", "B", "C", or "D") were displayed and the participant was instructed to press the spacebar if the current letter matched the one *N* images back. The participant had three runs (for *N*=1, 2, and 3, respectively). **Left panel:** Results of 1-Back. The run consists of 205=151+54 letters that did not match the previously displayed letter and 73=54+19 letters that matched the previously displayed letter. The participant correctly responded to all matched letters, but mistakenly pressed the spacebar for one unmatched letter (indicated by the edge $unmatched \rightarrow response$. The "unknown" node correctly indicates that the first displayed image in the file does not have a successor and so its matched state is unknown. **Middle panel:** 2-Back has similar behavior to 1-Back but the participant mistakenly pressed the spacebar for 17 unmatched images and failed to press the spacebar for 5 matched images. **Right panel:** 3-Back also has similar behavior, but far more errors. The participant pressed the spacebar 53 times after unmatched images.

Notes on data: The dataset had 143 *N*-Back event files containing a total of 48243 events. In the event files "large_H_small_" was incorrectly coded as "ignore". The following files were not included due to using a different encoding of events: *sub-4_ses-2*, *sub-5_ses-1*, and *sub-5_ses-2*. **Notes on the graph:** The back edges from responses to images were not displayed as they indicate transition to the next trial. The results for the entire dataset were similar, but there were a few additional low-count edges, indicating for example a participant pressing the spacebar multiple times.
